# Supplementary material for: Biosynthesis of Novel Ascorbic Acid Esters and Their Encapsulation in Lignin Nanoparticles as Carriers and Stabilizing Systems
Source: Int J Mol Sci. 2023 May 20;24(10):9044. doi: 10.3390/ijms24109044 (PMC10218833; doi:10.3390/ijms24109044)
Supplement: Supplementary file 1 [file ijms-24-09044-s001.zip › ijms-2392695-supplementary.pdf]

International Journal of *Molecular Sciences*

Article

Biosynthesis of novel ascorbic acid esters and their encapsulation in lignin nanoparticles as carriers and stabilizing systems

Eliana Capecchi [1], Davide Piccinino [1,*], Chiara Nascimben [1], Elisabetta Tomaino [1], Natalia Ceccotti Vlas [1], Sofia Gabellone [1], Raffaele Saladino [1]

[1] University of Tuscia, Department of Biological and Ecological sciences (DEB), Via San Camillo de lellis, 01100 Viterbo, Italy
[2] Istituto Romagnolo per lo Studio dei Tumori "Dino Amadori"; IRST-IRCCS, Via Piero Maroncelli, 40, 47014 Meldola FC
* Correspondence: saladino@unitus.it, d.piccinino@unitus.it

Citation: Capecchi, E.; Piccinino, D.; Nascimben, C.; Tomaino, E.; Vlas, N.C.; Gabellone, S.; Saladino, R. Biosynthesis of novel ascorbic acid esters and their encapsulation in lignin nanoparticles as carriers and stabilizing systems. *Int. J. Mol. Sci.* **2023**, *24*, 9044. https://doi.org/10.3390/ijms24109044

Academic Editors: Konstantin Volcho and Olga Luzina

Received: 25 April 2023
Revised: 12 May 2023
Accepted: 17 May 2023
Published: 20 May 2023

Copyright: © 2023 by the authors. Licensee MDPI, Basel, Switzerland. This article is an open access article distributed under the terms and conditions of the Creative Commons Attribution (CC BY) license (https://creativecommons.org/licenses/by/4.0/).

SI 1. Morphological analysis by FE-SEM images of biocatalyst **I**

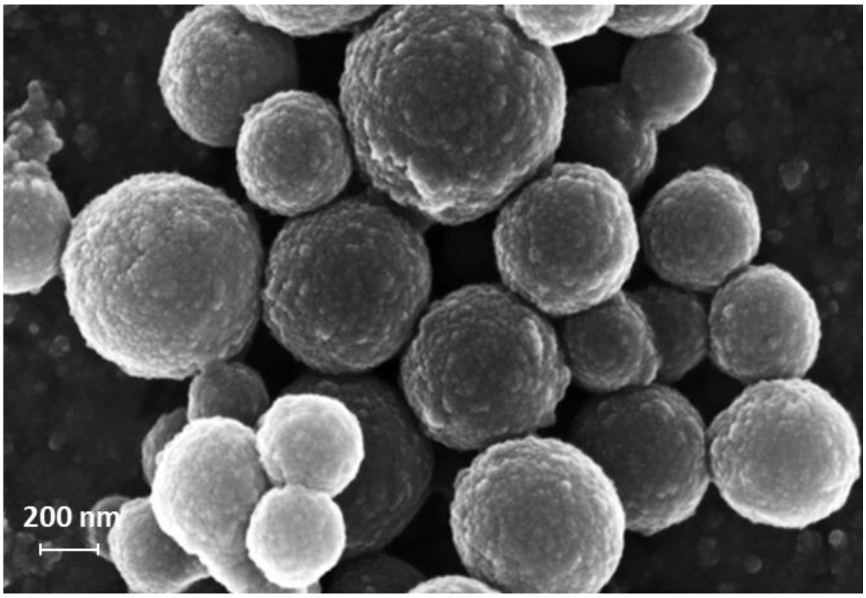

Figure S1. FE-SEM images of biocatalyst **I.**

SI 2. Synthesis protocol of ester **4** and **5**

The Mitsunobu synthesis of esters **4** and **5** was carried out via standard procedure in order to prepare reference samples. Briefly, a solution of the appropriate compound (1.0 eq.) in anhydrous THF (5.0 mL) and TPP (280 mg, 1.1 eq.) was cooled (0 °C) followed by addition of DIAD (1.0 eq.). After stirring for 48h at room temperature, the solvent was removed and the residue suspended in ethyl acetate. The organic phase was washed with saturated $NaHCO_3$ (15 mL x3), brine (15 mL x3), and dried by anhydrous Na_2SO_4.

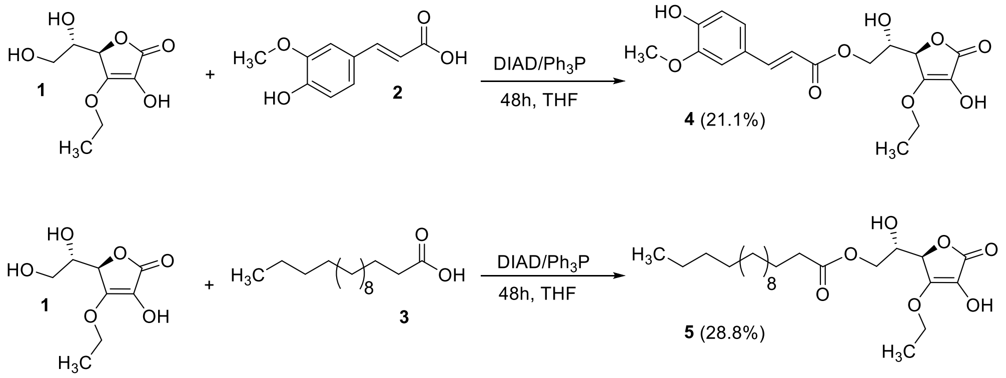

Figure S2. Synthesis ester **4** and **5** by Mitsunobu reaction.

SI 3. 1H and 13C NMR spectra of ester **4**

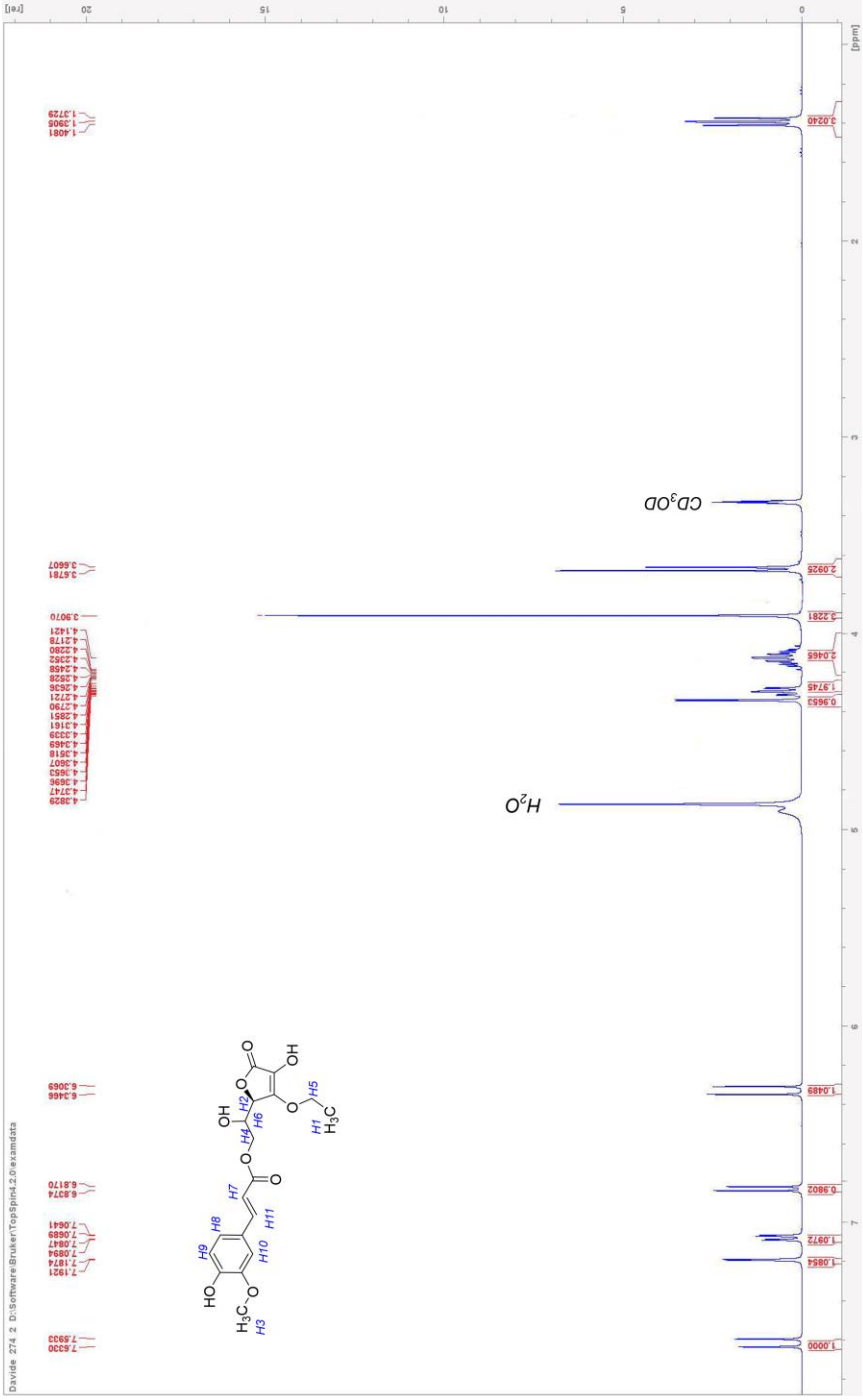

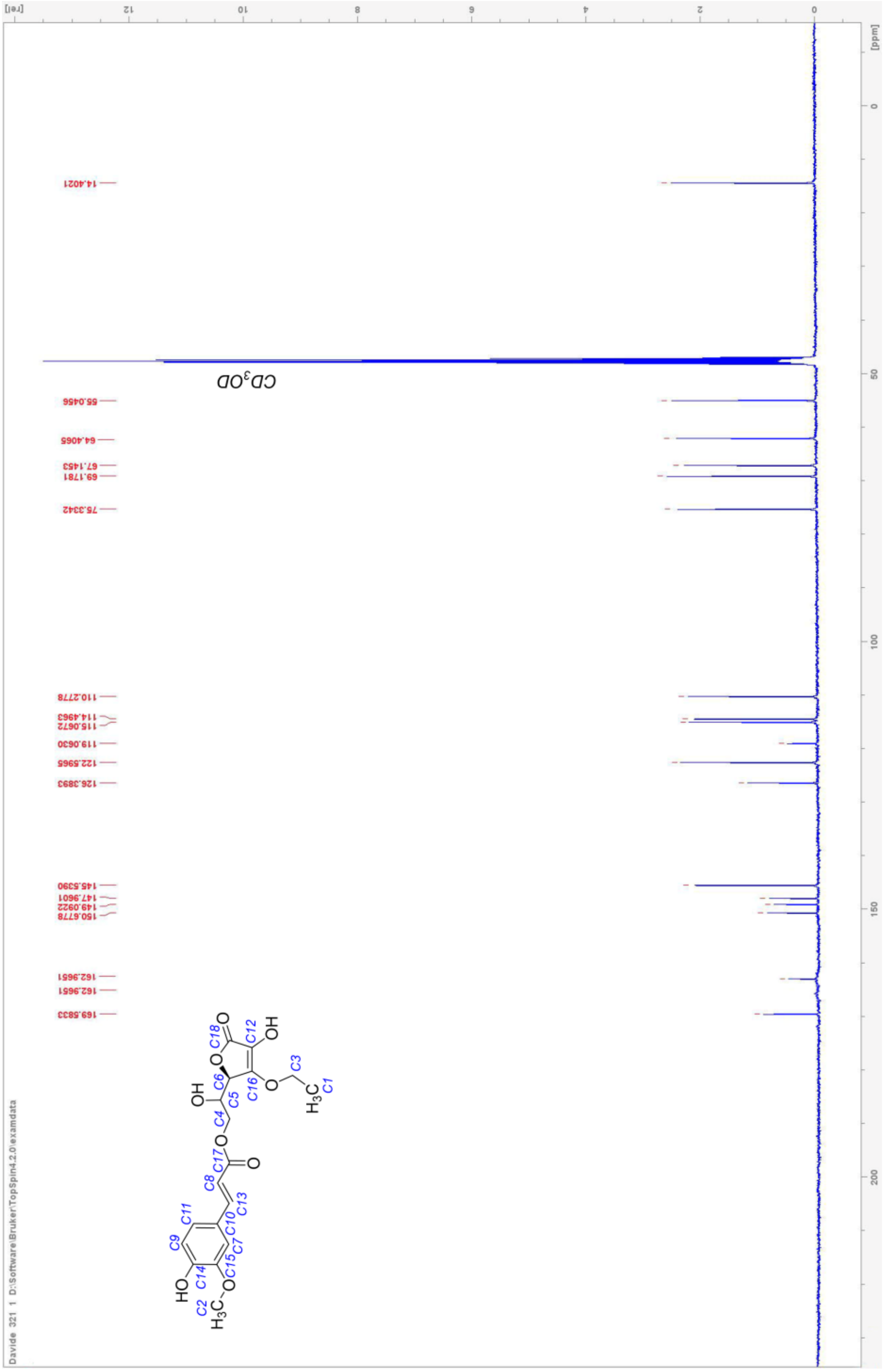

Figure S3. 1H and 13C NMR spectra of ester **4.**

SI 4. 1H and 13C NMR spectra of ester **5**

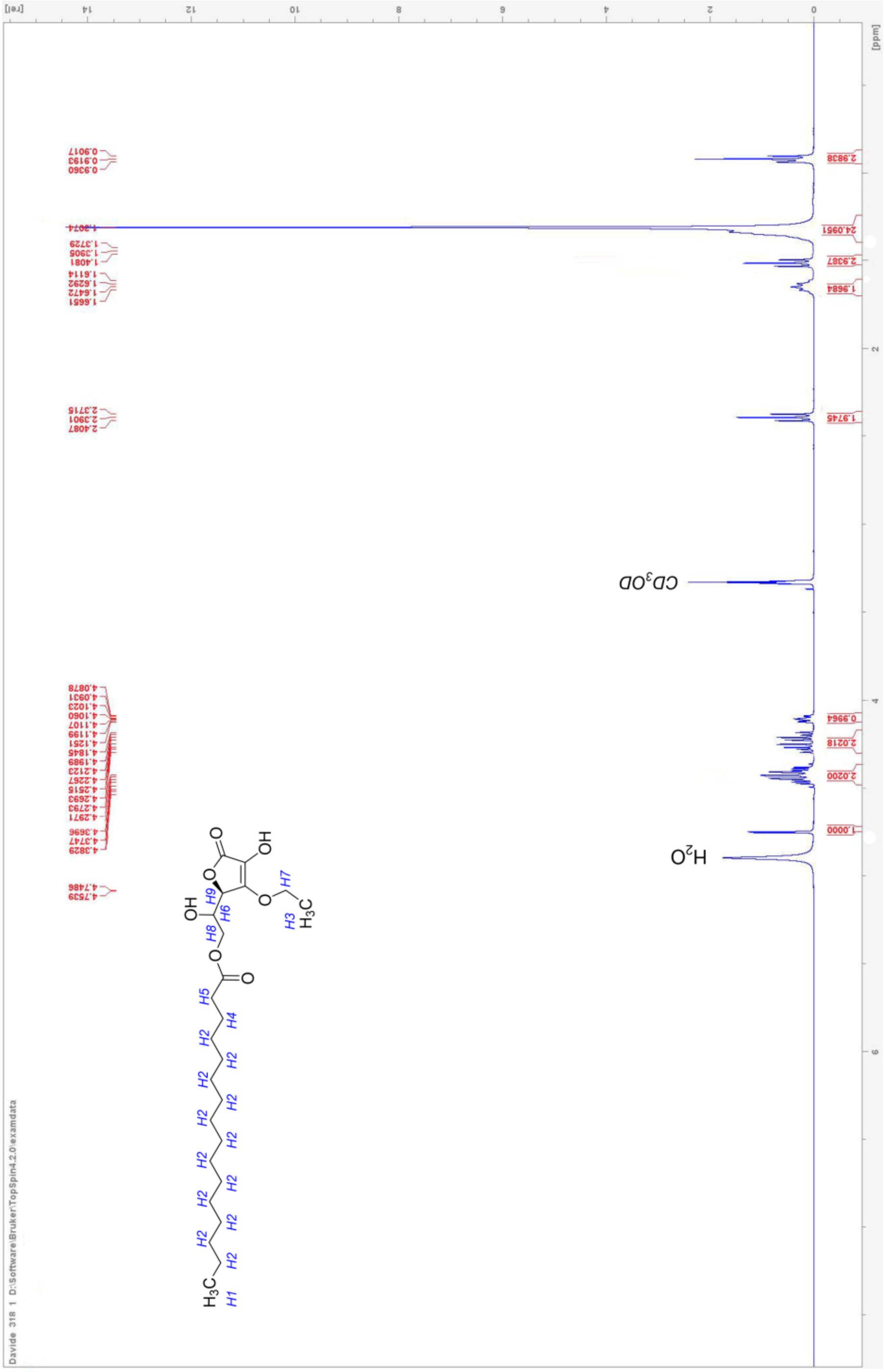

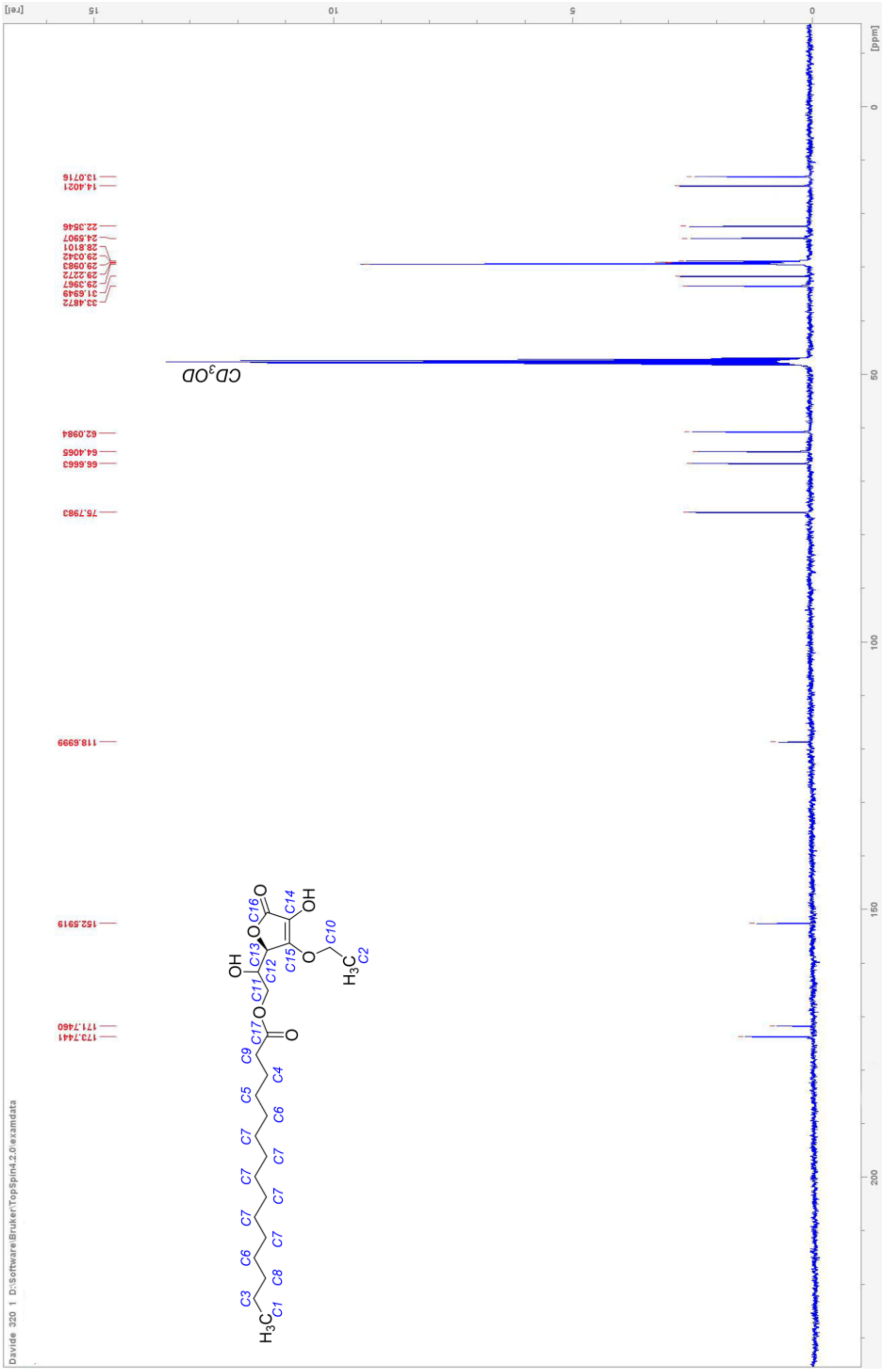

Figure S4. 1H and 13C NMR spectra of ester **5.**

SI 5. UV-vis analysis

The appropriate compound (1.0 µmol) was dissolved in milliQ water (2.8 mL) in the presence of MeOH (0.2 mL) and analyzed using quartz cuvette (3.0 mL, 1 cm path length) by Varian Cary UV 60 scan (Crawley, UK) in the range between 250 nm and 800 nm, with a scan speed of 400 nm/min and band width of 5 nm at 25°C under gentle stirring. The analysis was elaborated by the UV-Vis scan software.

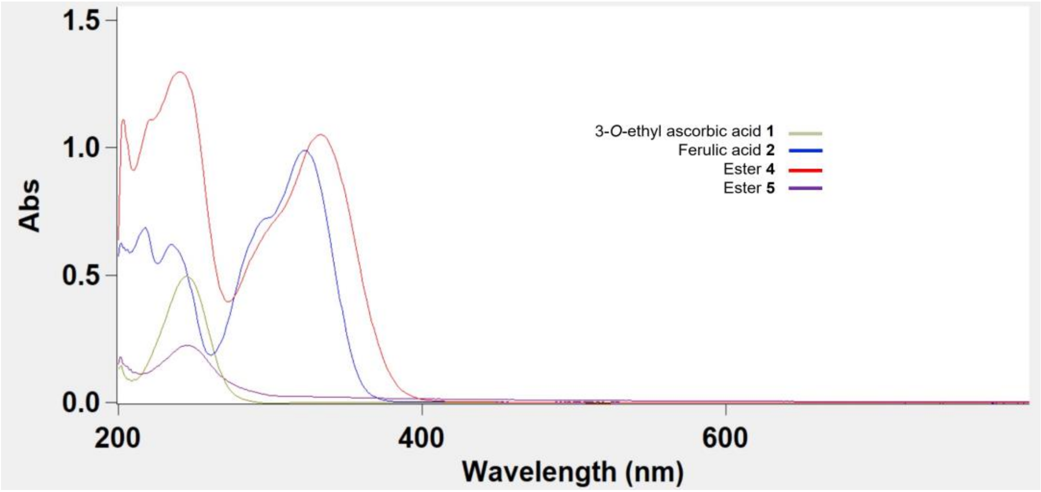

Figure S5. UV-vis scan of esters **4** and **5** compared with reference compounds (**1** and **2**).

SI 6. HPLC analysis

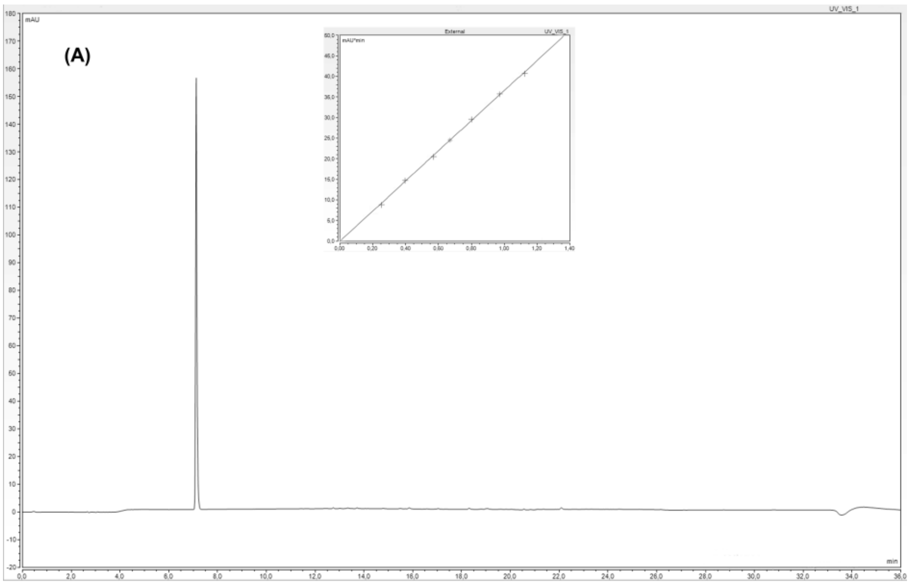

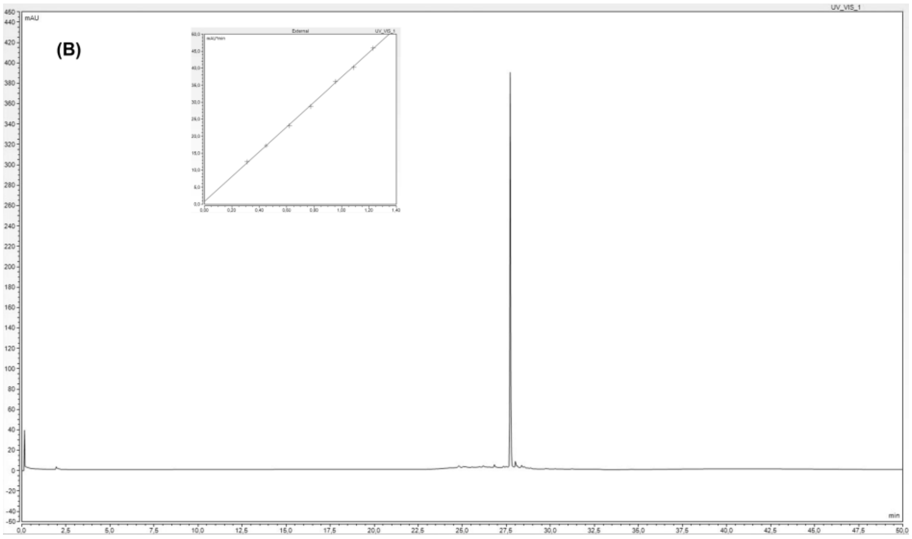

Figure S6. HPLC chromatograms of ester **4** (A) and **5** (B).

Disclaimer/Publisher's Note: The statements, opinions and data contained in all publications are solely those of the individual author(s) and contributor(s) and not of MDPI and/or the editor(s). MDPI and/or the editor(s) disclaim responsibility for any injury to people or property resulting from any ideas, methods, instructions or products referred to in the content.
